# Use of DNA‐alkylating pyrrole‐imidazole polyamides for anti‐cancer drug sensitivity screening in pancreatic ductal adenocarcinoma

**DOI:** 10.1002/cam4.5359

**Published:** 2022-10-19

**Authors:** Akiko Tsujimoto, Niina Matsuo, Xiaoyi Lai, Takahiro Inoue, Hiroyuki Yoda, Jason Lin, Yoshinao Shinozaki, Takayoshi Watanabe, Nobuko Koshikawa, Atsushi Takatori, Hiroki Nagase

**Affiliations:** ^1^ Division of Innovative Cancer Therapeutics Chiba Cancer Center Research Institute Chiba Japan; ^2^ Graduate School of Medical and Pharmaceutical Sciences Chiba University Chiba Japan; ^3^ Division of Cancer Genetics Chiba Cancer Center Research Institute Chiba Japan

**Keywords:** colorectal neoplasms, heterografts, imidazoles, pancreatic carcinoma, proto‐oncogene proteins p21(ras), pyrroles

## Abstract

**Background:**

Activating mutations of the *KRAS* occurs in >90% of pancreatic ductal adenocarcinoma (PDAC) cases. However, direct pharmacological targeting of the activated KRAS protein has been challenging. We previously reported that KR12, a DNA‐alkylating pyrrole‐imidazole polyamide designed to recognize the *KRAS* G12D/V mutation, showed an anti‐tumor effect in colorectal cancer. In this study, we evaluated the anti‐tumor effect of KR12 in PDAC.

**Methods:**

KR12 was synthesized by an automated peptide synthesizer PSSM‐8 and tested for anti‐tumor effect in PDAC mouse models.

**Result:**

KR12 inhibited tumor growth in a spontaneous PDAC mouse model, although the anti‐tumor activity appeared to be limited in a human PDAC xenograft model. We developed a pyrrole‐imidazole polyamide screening process based on the hypothesis that genetic elements otherwise unaffected by KR12 could exert attenuating effects on KRAS‐suppression‐resistant PDAC. We identified *RAD51* as a potential therapeutic target in human PDAC cells. A RAD51 inhibitor showed an inhibitory effect on cell growth and affected the cytotoxic activity of KR12 in PDAC cells.

**Conclusion:**

These data suggested that the simultaneous inhibition of RAD51 and mutant KRAS blockage would be an important therapeutic strategy for PDAC.

## Graphical abstract

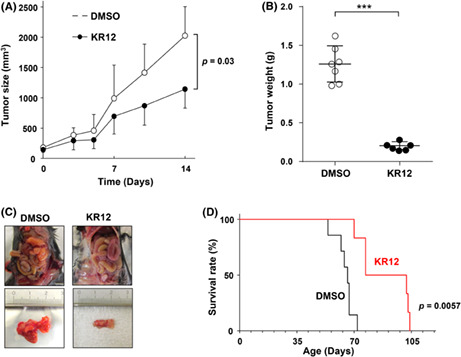

## 1 INTRODUCTION

Pancreatic ductal adenocarcinoma (PDAC) is a malignancy associated with high mortality rates. Although the combination of folinic acid/fluorouracil/irinotecan/oxaliplatin and nab‐paclitaxel plus gemcitabine have been standard as first‐line therapies, the survival rate of patients with PDAC remains poor.^1^, ^2^ Remarkable progress has been achieved toward the development of molecular targeted therapy and immunotherapy for various types of cancers. However, the benefits of these therapeutic regimens in patients with PDAC are limited.^3^, ^4^ In PDAC, the mutagenic event of *KRAS* occurs at a frequency of approximately 95%, with the majority observed in codon 12 D/V. This is perhaps one of the most frequently mutated oncogenes in a number of different cancer types and a key molecular switch for regulating critical cellular processes, such as proliferation, differentiation, and survival.^5^, ^6^ Recent reports also suggested that KRAS G12C inhibitors can suppress tumor growth by inhibiting KRAS‐dependent signaling upon covalent binding at Cys12.^7^, ^8^ However, with the sole exception of G12C, most Ras species have relatively smooth surfaces with few solvent‐accessible crevices for the binding of small molecule inhibitors.^9^ This renders the strategy of directly targeting activated G12D/V KRAS a great pharmacological challenge.

Investigators have faced difficulty in inhibiting KRAS at the protein level. Therefore, we recently developed a novel approach targeting *KRAS* at the nucleotide level to prevent the transcription of constitutively active *KRAS* species, using hairpin‐structured *N*‐methylpyrrole (Py)‐*N*‐methylimidazole (Im) polyamides (PI polyamides). PI polyamides can be designed to bind, with high affinity, the minor groove of DNA helices containing the motif of interest. Through this method, Py subunits preferentially recognize thymine, adenine, and cytosine, and Im subunits can selectively interact with guanines.^10^ We previously synthesized KR12, a DNA‐alkylating PI polyamide that selectively binds to the *KRAS* G12D/V mutation sequence (WCGCCWWCA; W denotes weak bases A or T). We found that KR12 selectively bound mutant *KRAS* and led to observable anti‐tumor effects in colorectal cancer models,^11^ most likely due to the induction of cell death as a consequence of mutant *KRAS* silencing. These results prompted us to investigate the anti‐tumor activity of KR12 in PDAC harboring the *KRAS* G12D/V mutation. In addition, throughout the course of our investigations, we also devised a multi‐component PI polyamide screening process that led to the discovery of a potential key element in susceptibility to KR12.

## 2 MATERIALS AND METHODS

### 2.1 Compound synthesis

For our initial experiment, we synthesized KR12 as previously described.^11^ For subsequent investigations, we designed and synthesized CCC‐002 and CCC‐003 based on revised synthetic steps as previously reported.^12^ The reagents used in compound synthesis were shown in Table S1. We used a stepwise solid phase reaction on a semi‐automated peptide synthesizer, PSSM‐8 (Shimadzu) (scale: 10 μmol)^13^, ^14^, ^15^ as the basis for the synthesis of the PI polyamide backbone. The synthetic steps for the alkylating moiety indole‐*seco*‐CBI have been previously reported.^16^ HPLC LC‐20 (Shimadzu) with a 10 × 150 mm Phenomenex Gezmini‐NX3u 5‐ODS‐H reverse‐phase column was used for purification (A: 0.1% acetic acid in Millipore milliQ water; B: acetonitrile, flow rate 10 ml/min). CCC‐003 was synthesized by the amide coupling of the N‐acetylated polyamide backbone (Ac‐NH‐PyPyPyPyβPyPyPy‐γ‐ImImβImImPy‐COOH) with 3.5 eq of indole‐seco‐CBI using 3.5 eq of WSC as the coupling reagent in NMP at room temperature for 12 h. After reaction, the conjugate was purified by HPLC (43% isocratic mode, acetonitrile, and 0.1% hydrochloric acid in Millipore milliQ water at 340 nm peak observed at 28.0–29.5 min). The purified product was reconstituted in DMSO. Liquid chromatography–mass spectrometry characterization: [M + H]^+^ *m*/*z* calcd for C_102_H_105_ClN_34_O_18_ = 2128.80, found 2129.10.

### 2.2 Murine models of human PDAC

We received approval from the animal care and ethics committee of Chiba Cancer Center Research Institute prior to conducting the in vivo experiments. For the xenograft experiment, female BALB/c nude mice (4–6 weeks old; Charles River Laboratories) were subcutaneously injected with KP‐4 cells (2 × 10^6^ cells). After the tumor size reached 75–100 mm^3^, the mice were intraperitoneally injected with DMSO or KR12 (3 mg/kg/week) once weekly for 4 weeks (*n* = 6/group). The tumor size was measured by a caliper and calculated using the formula V = L × W × H × π /6. The mice were sacrificed 28 days after the initial treatment or after the tumor size reached 2000 mm^3^. The tumors and organs were fixed in 4% paraformaldehyde. Sections (4 μm) were deparaffinized by immersing in xylene and rehydrated, followed by immunostaining with anti‐RAD51 (ab133534; abcam) antibody.

Genetically engineered mouse lines were created as described by Ijichi et al.^17^ Briefly, Tgfbr2flox/flox mice (C57BL/6)^18^ were mated with Ptf1acre/+ mice (C57BL/6.DBA/2)^19^ to generate Ptf1acre/+;Tgfbr2flox/flox mice (C57BL/6.DBA/2). After the initial cross, Ptf1acre/+;Tgfbr2flox/flox, and LSL‐KRASG12D/+;Tgfbr2flox/flox mice (C57BL/6.129/SvJae) were backcrossed for >10 generations to produce C57BL/6 inbred mice and crossed to obtain Ptf1acre/+;LSL‐KRASG12D/+;Tgfbr2flox/flox (PKF) and Tgfbr2flox/flox control mice. The endogenous KRASG12D expression plus Tgfbr2 knockout genetically engineered mice received an intraperitoneal injection of 34 nmol KR12 (3 mg/kg body weight, 1.25% DMSO in PBS) or 1.25% DMSO in PBS weekly after the initial injection at postnatal day 35 to evaluate the survival time and histology. Mice check‐ups were performed daily by the veterinary staff, and qualified veterinary physicians performed medical check‐ups on a monthly basis. Tumor‐bearing mice were euthanized when considered moribund or the tumor diameter reached 2 cm. The pancreas of mice meeting the condition for euthanization and sacrifice was excised and measured for histological examination of the tumors.

### 2.3 Cell culture

Human PDAC KP‐4, PANC‐1, Capan‐1, and AsPC‐1 cells were maintained in DMEM/F12, DMEM, Iscove's Modified Dulbecco's Medium, and RPMI, respectively. KP‐4 and PANC‐1 were obtained from the ATCC Cell Bank, while Capan‐1 and AsPC‐1 were obtained from the RIKEN Cell Bank. Mouse PDAC cell lines K375 and K399 obtained from an endogenous *KRAS* G12D expression plus *Tgfbr2* knockout mouse^7^ were maintained in RPMI 1640 medium in collagen‐coated dishes. All cell lines were supplemented with 10% FBS (Thermo Fisher Scientific) and penicillin/streptomycin (Thermo Fisher Scientific) and cultured in a humidified atmosphere at 37°C with 5% CO_2_.

### 2.4 Cell proliferation assay

Cells were seeded in triplicate in a 96‐well plate at a concentration of 3000 cells/well and treated with increasing concentrations of KR12 or RAD51 inhibitor B02 (Cayman Chemical). And 0.1% DMSO was used as control. At 72 hours after treatment, WST assay using the CCK‐8 (Fujifilm‐Wako Chemicals) was performed to determine cell proliferation.

### 2.5 Real‐time RT‐PCR

Total RNA was prepared using the RNeasy Plus Mini kit according to the instructions provided by the manufacturer (Qiagen). RNA (0.5 μg) was reverse transcribed to cDNA via SuperScript VILO cDNA Synthesis System (Invitrogen). The obtained cDNA was used for quantitative PCR (PowerUp SYBR Green Master Mix, Thermo Fisher Scientific). Three independent measurements were performed, and the expression values were normalized to those of *RPS18*. Quantitation of gene expressions was performed using standard curve method. Serial dilutions of cDNA derived from KP4 cells were used as template for the standard curve. The PCR primer sequences used were as follows: human *KRAS*, 5′‐GGAGAGAGGCCTGCTGAA‐3′ and 5′‐TGACCTGCTGTGTCGAGAAT‐3′; human *RAD51*, 5’‐TTTGGCCCACAACCCATTTC‐3′ and 5′‐TTAGCTCCTTCTTTGGCGCA‐3′; human *RPS18*, 5′‐GAGGATGAGGTGGAACGTGT‐3′ and 5’‐TCTTCAGTCGCTCCAGGTCT‐3′;mouse *Kras*, 5’‐CAAGAGCGCCTTGACGATACA‐3′ and 5′‐CCAAGAGACAGGTTTCTCCATC‐3′; mouse *Rad51*, 5′‐GCGCCGGTCAGAGATCATAC‐3′ and 5′‐TGGCATGTAACAGCCAACGTA‐3′; mouse *Rps18*, 5′‐TCCCTGAGAAGTTCCAGCAC‐3′ and 5′‐ CCACATGAGCATATCTCCGC‐3′.

### 2.6 Western blotting analysis

KR12‐treated cells were lysed using RIPA buffer containing phosphatase and complete proteinase inhibitor. BCA protein assay kit (Thermo Fisher Scientific) was used to determine protein concentration. Proteins were separated by SDS‐PAGE and transferred to PVDF membranes. The membranes were blocked with 5% skimmed milk for 60 min at room temperature and subjected to immunoblotting using the primary antibodies of anti‐KRAS (sc‐30; Santa Cruz Biotechnology), anti‐RAD51 (ab133534; abcam) and anti‐GAPDH (016–25,523; Wako). The results were quantified using the ImageJ software.

### 2.7 Silencing *KRAS* G12D

*KRAS* G12D siRNA corresponding to codon 12 was designed and synthesized as previously described.^18^ siRNA of hKRAS#3GAT for KRASG12D (sense sequence 5′‐GUUGGAGCUGAUGGCGUAG‐3′TT and antisense 5′‐CUACGCCAUCAGCUCCAAC‐3′TT) were obtained from Sigma Genosys. The control siRNA was siControl MISSION siRNA (SIGMA GENSYS). PDAC cells were forward‐transfected with siRNA using Lipofectamine RNAiMAX (Thermo Fisher Scientific). Briefly, we diluted the siRNA into Opti‐MEM (Thermo Fisher Scientific) to a final concentration of 30 pM; 2.5 ml of the mixture was added to each well of a six‐well plate for incubation. Cells (1 × 10^5^/well) were incubated for 48 h at 37°C prior to gene knockdown assay by quantitative real‐time RT‐PCR. For cell growth assays, 90 μl of 1 pM siRNA in Opti‐MEM was aliquoted into each well of a 96‐well plate. Cells (1 × 10^4^/well) were monitored for 48 h using the IncuCyte Live Content Imaging System (Essen Bioscience); cell growth was evaluated by the accompanying live imaging analysis software.

### 2.8 Comparative expression profiling

KP‐4 cells (10^4^–10^5^) were plated overnight prior to the administration of polyamide (100 nM) for 12 h. DMSO (0.01%) was used as control. Cells were lysed for RNA extraction using the RNeasy Plus Mini Kit (Qiagen), and sample replicates were labeled with the RNA Spike‐In Kit to be analyzed by SurePrint G3 Human GE 8 × 60K V2 expression microarrays (Agilent). Differential expression analyses were performed with our previously developed custom R workflow.^20^ We initially screened for the bottom 20‐percentile downregulated candidates (log2FC <0) with *P*‐values below the predefined significance level (*p* < 0.0001). We isolated those that were affected only by CCC‐002 and CCC‐003, as these polyamides did not directly indicate *KRAS* mutation. We subsequently narrowed the search parameters to include only the candidates associated with cancer‐related pathways using information from Kyoto Encyclopedia of Genes and Genomes.^21^ We confirmed that the intragenic regions contained binding sites for the respective polyamides, and that the elevated expression levels of these genes affected survival based on records available in the R2 Genomics Analysis and Visualization Platform.^22^ Among the final candidates, we selected RAD51 for further evaluation. The microarray data are deposited at the Gene Expression Omnibus (GEO) repository under accession number GSE208243.

The expression level of *RAD51* in pancreatic adenocarcinoma and normal pancreatic tissues was compared by analyzing datasets from TCGA and GTEx via the Gene expression profiling interactive analysis (GEPIA).^23^

### 2.9 Statistical analysis

Statistical analyses were performed using the GraphPad Prism 6.0 software. Unpaired *t*‐test was used to determine the difference between two groups. One‐way ANOVA followed by Holm–Sidak's multiple comparison test or two‐way ANOVA followed by Tukey's multiple comparison test were performed for multiple group analysis. For the mouse xenograft studies, repeated measures ANOVA followed by the Bonferroni post test were used to evaluate the difference in tumor volume between the groups. *p* Values less than 0.05 were considered statistically significant.

## 3 RESULTS

### 3.1 KR12 suppresses tumor progression in murine PDAC models

**FIGURE 1:**
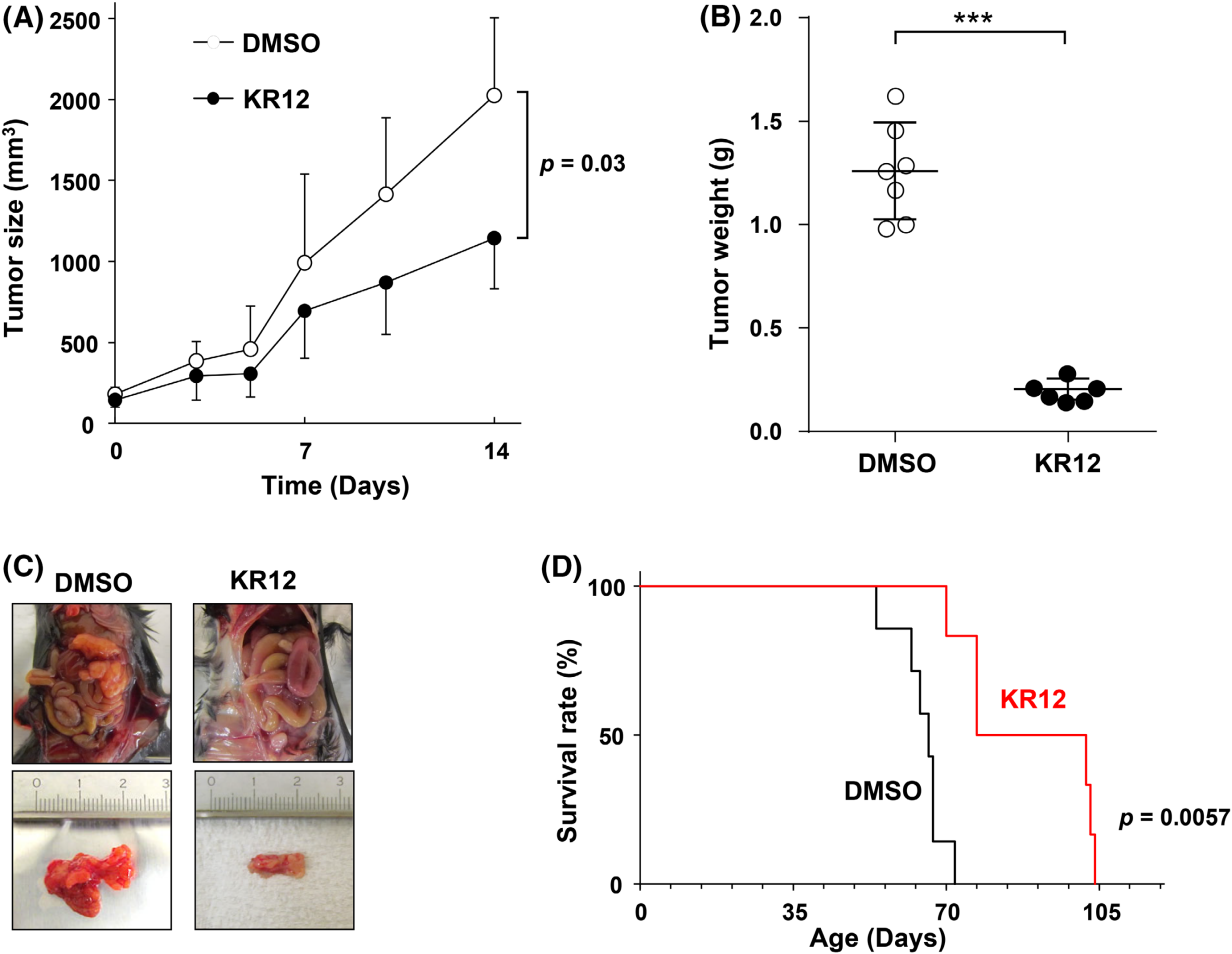
Treatment with KR12 exhibits anti‐tumor activity in mouse models of PDAC. (A) KR12 suppresses tumor growth in the xenograft model using KP‐4 cells. KR12 (3 mg/kg body weight) was intraperitoneally injected weekly. The administration of KR12 was initiated after the average tumor size reached 100 mm^3^. Tumor volume and body weight of the mice were measured twice weekly. The *p*‐value was determined by repeated measures ANOVA followed by the Bonferroni post test (*p < 0.05). Data are presented as the mean ± SD. (B–D) Treatment with KR12 reduced tumor growth and prolonged survival in PKF mice. KR12 (3 mg/kg body weight) was intraperitoneally administered once weekly from birth to 35 days. (B) The tumor weight ratios of the KR12 and DMSO treatment groups are depicted by dot plots and the data are represented as the mean ± SD. The *p*‐value was determined using the unpaired *t*‐test (***p < 0.001). (C) Images showing mice and tumors from the KR12 and DMSO treatment groups at the time of dissection. (D) Kaplan–Meier plots of overall survival in PKF mice. The *p*‐value was determined using the two‐sided log‐rank test.

We first evaluated the anti‐tumor potential of KR12 in murine models of PDAC. In a xenograft mouse model using human PDAC cells, intravenous administration of KR12 significantly retarded the tumor growth versus treatment with DMSO (Figure 1A and Figure S1A). The anti‐tumor activity of KR12 was also confirmed for *KRAS*‐driven pancreatic tumors in PKF mice, with no observable loss in body weight (Figure 1B,C; Figure S1B). The survival of KR12‐treated mice was significantly prolonged compared with that of mice receiving DMSO (Figure 1D). Pathological examination revealed that tumors in the control group exhibited consistently well‐differentiated glandular architecture with progressive invasion of tumor cells into the stroma, occupying the entire pancreas. This resulted in almost complete loss of normal pancreatic tissue. Atypical glandular cells and pancreatic intraepithelial neoplasia were found in ductal epithelial tissues. Infiltration of granulocytes into surrounding pancreatic tissues was also observed, suggesting the development of progressive pancreatitis. Pancreatic tissues obtained from the KR12‐treated group showed sporadic development of well‐differentiated PDAC, as well as atypical glandular cells and pancreatic intraepithelial neoplasia, surrounded by fibroblastic stroma and largely retained non‐neoplastic pancreatic acinar cells. These data suggested that the pancreatic tissues and endocrine glands in the Langerhans islets largely remained normal. Beyond the pancreas, we did not observe pathological damage to other organs, including the liver and kidneys (Figure S2).

### 3.2 Resistance to KR12 varies among PDAC cells harboring the *KRAS* G12D/V mutation

**FIGURE 2:**
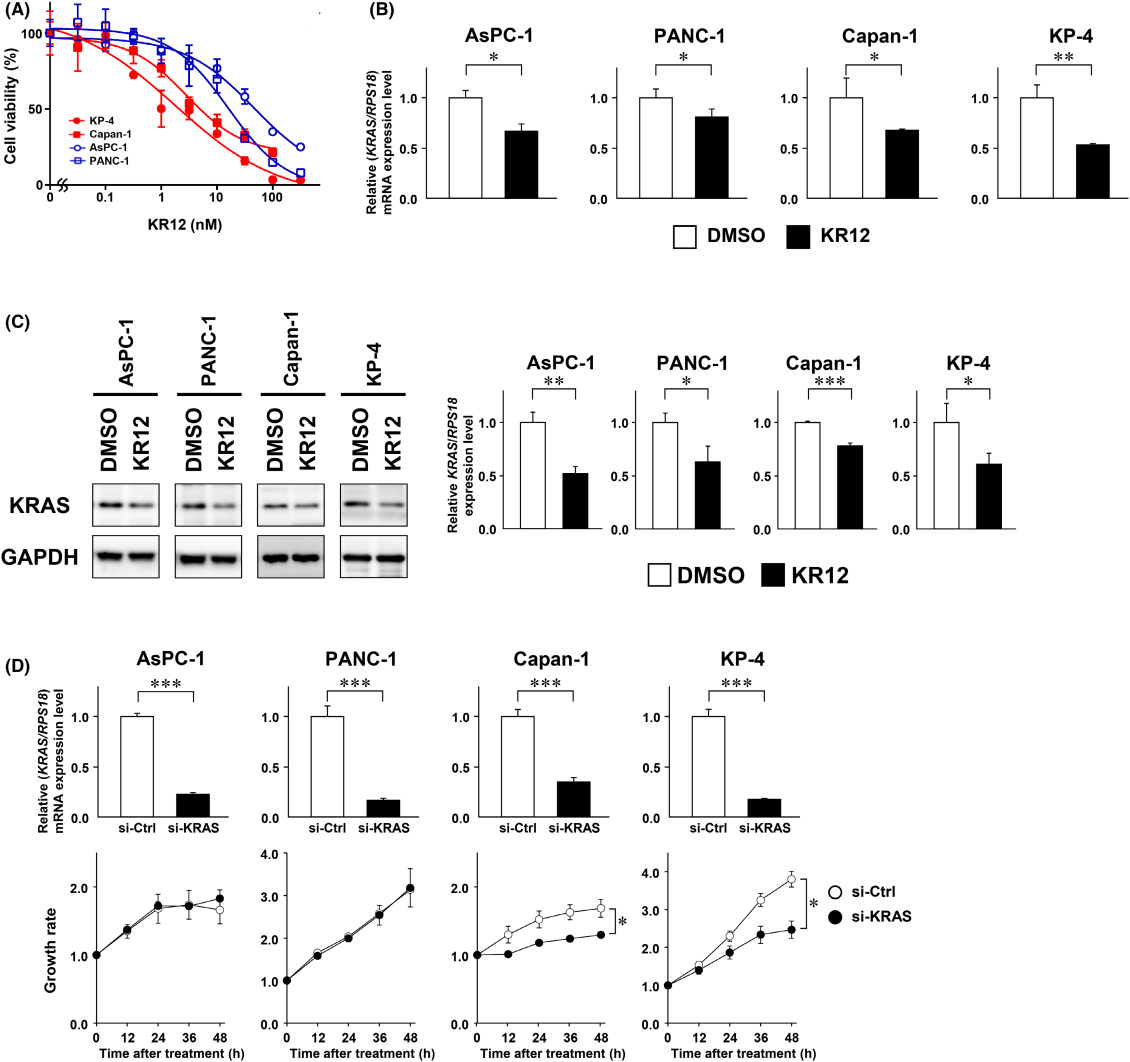
KR12 suppresses the proliferation of KRAS‐dependent human PDAC cells. (A) WST assays were performed to examine the cell viability at 72 h after treatment with KR12 (0.1–1000 nM) in human PDAC cells. (B, C) After treatment with KR12 (100 nM) for 48 h, the expression levels of KRAS mRNA (B) and protein (C) were analyzed by quantitative RT‐PCR and western blotting, respectively. Data are represented as the mean ± SD. The *p*‐values (*p < 0.05; **p < 0.01; ***p < 0.001) were determined using the unpaired *t*‐test. (D) Human PDAC cells were transiently transfected with siKRAS or control siRNA (10 nM), and cell growth was monitored by a real‐time cell imaging system for 48 h. Expression levels of *KRAS* were confirmed by quantitative RT‐PCR.

**FIGURE 3:**
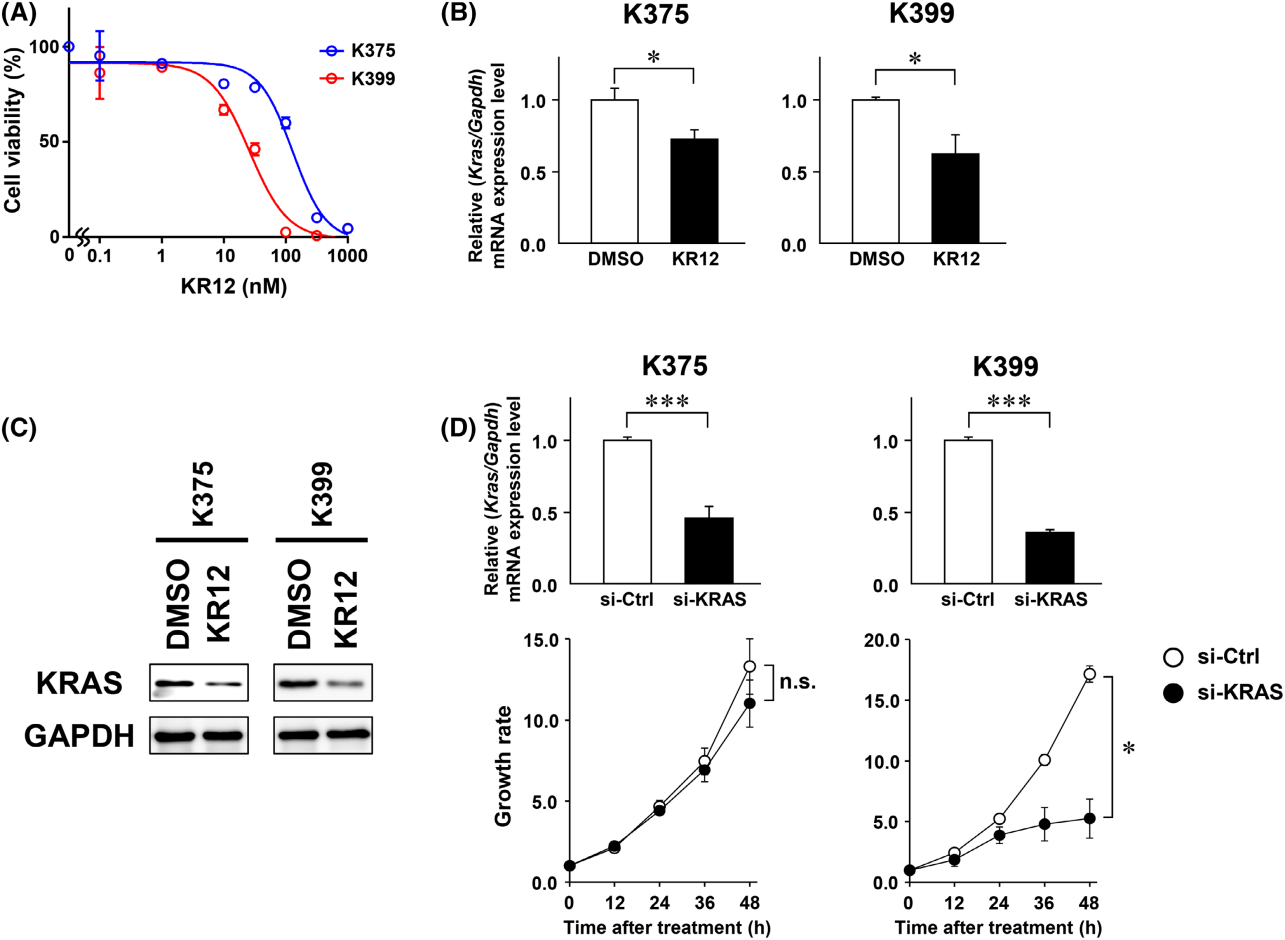
The anti‐proliferative effect of KR12 differs between the cell lines established from the tumor tissues of PKF mice. (A) WST assays were performed to examine the cell viability at 48 h after treatment with KR12 (0.1–1000 nM) in K375 and K399 cells. (B, C) After treatment with KR12 (3–300 nM) for 48 h, the expression levels of KRAS mRNA (B) and protein (C) were analyzed by quantitative RT‐PCR and western blotting, respectively. Data are represented as the mean ± SD. The *p*‐value was determined using the unpaired *t*‐test (*p < 0.05). (D) K375 and K399 cells were transiently transfected with siKRAS or control siRNA (100 nM), and cell growth was monitored by a real‐time cell imaging system for 48 h. Knockdown of *KRAS* expression was confirmed by quantitative RT‐PCR. Data are represented as the mean ± SD. The *p*‐value was determined using the unpaired *t*‐test (***p < 0.001) and repeated measures ANOVA followed by the Bonferroni post test (*p < 0.05). n.s., not significant.

As complete response to KR12 was not observed in the KP‐4 human pancreatic cancer xenografts, we investigated the anti‐proliferative effect of KR12 in four human PDAC cells harboring *KRAS* G12D/V. WST assays demonstrated that KP‐4 (G12D/WT) and Capan‐1 (G12V/G12V) cells were sensitive to KR12 (IC_50_ values of 3.6 and 2.8 nM, respectively). In contrast, AsPC‐1 (G12D/G12D) and PANC‐1 (G12D/WT) cells were relatively resistant to KR12 (IC_50_ values of 88.1 and 42.5 nM, respectively) (Figure 2A), even though KR12 significantly downregulated the expression of KRAS at the mRNA and protein levels in all four cell lines (Figure 2B,C). To test whether *KRAS* suppression is sufficient to impair cell growth, the PDAC cells were transiently transfected with siRNA against G12D or G12V‐mutated *KRAS*. The knockdown of *KRAS* expression suppressed cell proliferation in Capan‐1 and KP‐4 cells, but not in AsPC‐1 and PANC‐1 cells (Figure 2D). Similar results were obtained in two types of cell lines established from the tumor tissues of PKF mice. Although KRAS expression was suppressed in both cells, treatment with KR12 significantly inhibited the growth of K399 cells, but not that of K375 cells (Figure 3A–C). The suppression of cell proliferation by siRNA‐mediated knockdown of *KRAS* expression was observed in K399 cells, but not in K375 cells (Figure 3D). These data suggested that KRAS dependency is lower in PDAC cells than colorectal cancer cells in vivo. Hence, additional therapeutic targets are needed to improve the efficacy of KRAS‐targeted therapy.

### 3.3 PI polyamides with different DNA sequence recognition identify a potential therapeutic candidate gene in PDAC cells

**FIGURE 4:**
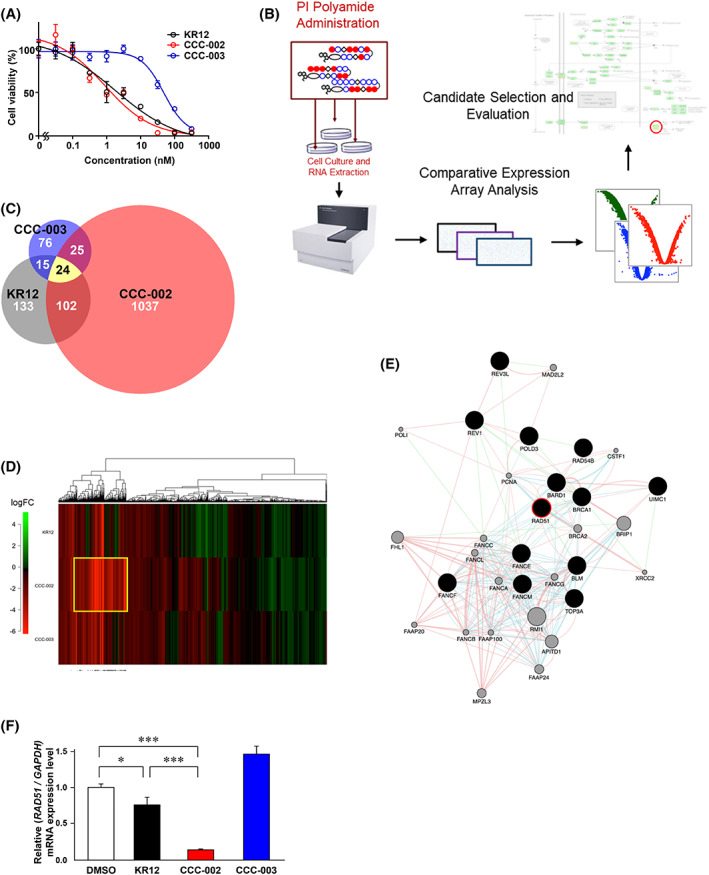
Microarray analysis using PI polyamides with different DNA sequence recognition. (A) WST assays were performed to examine the cell viability at 72 h after treatment with KR12, CCC‐002, and CCC‐003 (0.1–1000 nM) in KP‐4 cells. (B) Scheme for multi‐component PI polyamide screening by expression profiling to identify key elements involved in KR12‐resistant PDAC. (C) Venn diagram of the 20‐percentile of most downregulated genes identified from the PI polyamide screening, with each circle denoting genes from the respective group for each polyamide. (D) Heat map of the three‐component PI polyamide profiling; the color scale from red to green indicates normalized log_2_FC. Red, downregulated (log_2_FC <0); green, upregulated (log_2_FC >0). The yellow boxed region denotes genes that were considered initial screening targets for further analysis. (E) Interaction network between some of the major genes (black closed circles). Pink, physical interaction; purple, co‐expression; orange, predicted interactions; blue, co‐localization; cyan, pathway‐based interactions; green, genetic interactions; and yellow, shared protein domains. Network revisualized in Cytoscape. (F) After treatment with KR12, CCC‐002, and CCC‐003 (100 nM) for 12 h, the expression levels of RAD51 mRNA were analyzed by quantitative RT‐PCR.

We next performed screening experiments with the other two PI polyamides to identify a potential therapeutic target gene against PDAC. This investigation was based on the hypothesis that certain genetic elements unaffected by the binding of KR12 may have modulated the effect of the polyamide, leading to the presentation of non‐KR12‐susceptible characteristics in human cancer cell lines. We selected CCC‐002 and CCC‐003 which were previously designed to contain specific binding sequences (WGGCWCCCA and WGGWGGWWWA, respectively) and showed anti‐proliferative effects in neuroblastoma cells. These two compounds have no binding ability to the sequence of mutant *KRAS*. Following treatment of KP‐4 cells with three compounds, CCC‐002 showed a comparable anti‐proliferative effect to that observed with KR12 (Figure 4A). To identify a target gene of CCC‐002, we performed microarray and pathway analyses (Figure 4B–E). The initial screening yielded 113 genes based on the top 10 enriched pathways. We subsequently checked whether these genes contained a binding sequence for CCC‐002 in their coding region, and found 91 genes to be possible CCC‐002 targets for further investigation. Kaplan–Meier survival curves indicated a poorer survival for patients with PDAC that expressed high levels of nine genes; these genes were considered potential candidates for CCC‐002 target genes (Figure S3). Among those, we focused on *RAD51*, as its role in carcinogenesis and drug resistance had yet to be fully explored. The expression level of *RAD51*, analyzed using GEPIA, was higher in pancreatic adenocarcinoma tissues compared to normal pancreatic tissues (Figure S4). All PDAC cells used in this study and KP‐4 tumor tissues showed RAD51 expression (Figure S5). Quantitative PCR analysis confirmed that CCC‐002 markedly downregulated the mRNA expression of *RAD51* in KP‐4 cells (Figure 4F), while KR12 treatment did not downregulate the *RAD51* expression in cultured PDAC cells and KP‐4 tumor tissues (Figure S5). In addition, RAD51 expression was not altered by inhibition of KRAS signaling pathway (Figure S5). Together, these findings implicated that KRAS is not involved in a transcriptional regulation of *RAD51*.

### 3.4 Combination of KR12 and RAD51 inhibitor enhances the anti‐tumor effect

**FIGURE 5:**
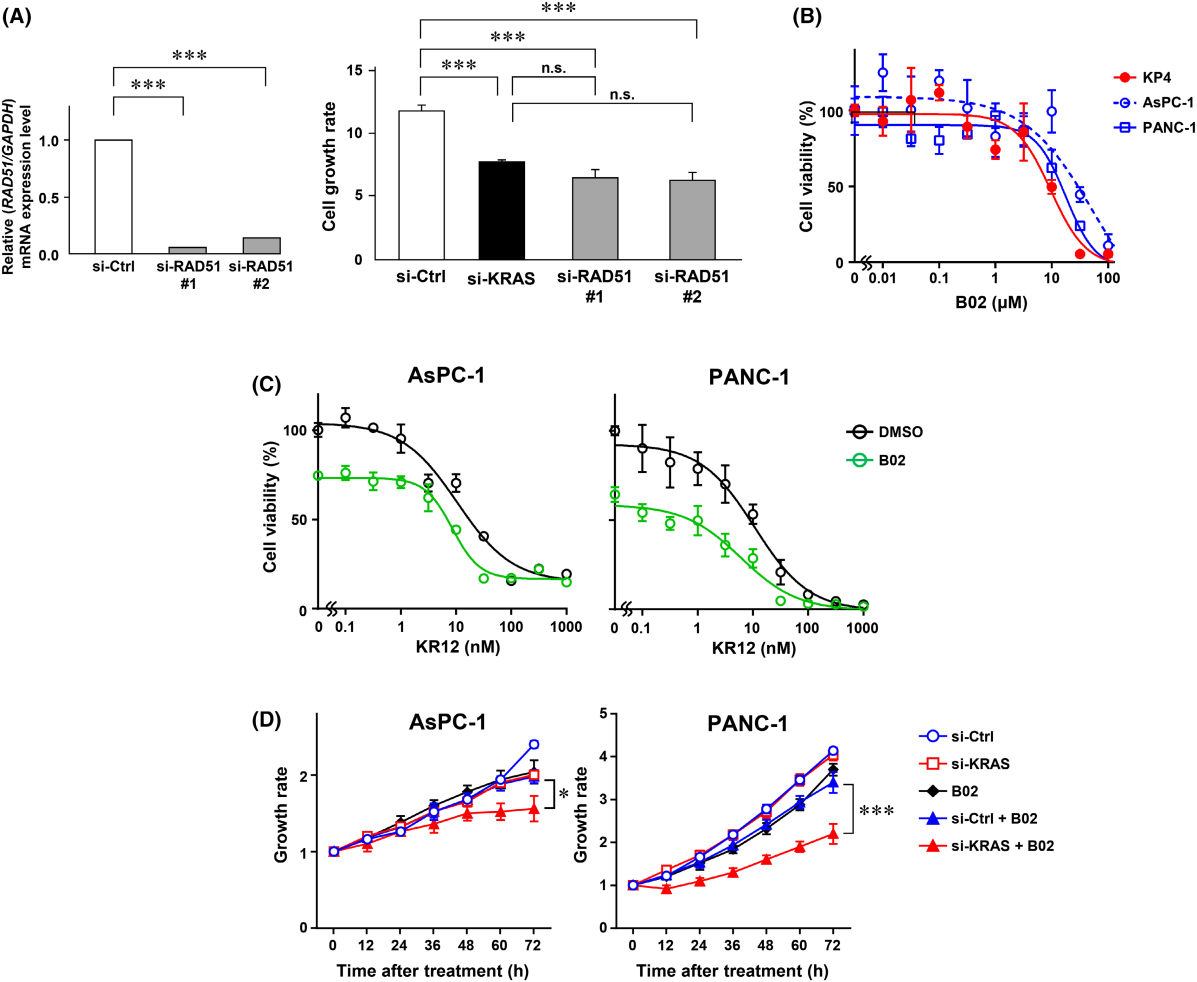
RAD51 inhibitor shows a combinatory effect with KR12 and knockdown of *KRAS* in PDAC cells. (A) KP‐4 cells were transfected with siKRAS or control siRNA (10 nM), and quantitative RT‐PCR was performed to determine *KRAS* expression was at 48 h after transfection. The cell growth rate was monitored by a real‐time cell imaging system for 48 h. Data are presented as the mean ± SD. The *p*‐value was determined by one‐way ANOVA followed by Holm–Sidak's multiple comparison test (***p < 0.001). n.s., not significant. (B) WST assays were performed at 72 h after B02 treatment (0.01–100 μM). (C) AsPC‐1 and PANC‐1 cells were treated with B02 (10 μM) and increasing concentrations of KR12. The inhibition of cell growth was determined by WST assays. Data are presented as the mean ± SD. (D) AsPC‐1 and PANC‐1 cells were transiently transfected with siKRAS (10 nM). At 24 h after transfection, the cells were treated with B02 (3 μM), and cell growth was monitored by a real‐time cell imaging system for 72 h. Data are presented as the mean ± SD, and the *p*‐values were determined using two‐way ANOVA followed by Tukey's multiple comparison test.

To test whether blockage of RAD51 may be a therapeutic strategy against PDAC, siRNA against RAD51 was introduced into KP‐4 cells. As shown in Figure 5A, silencing of RAD51 expression decreased the cell proliferation; this effect was comparable to that observed after knockdown of *KRAS* expression. Previous studies have demonstrated that the small molecule compound B02 inhibits the activity of RAD51 and sensitizes breast cancer cells to DNA‐damaging agents.^24^, ^25^ Thus, we examined the anti‐proliferative effect of B02 in PDAC cells. B02 inhibited the growth of KP‐4, AsPC‐1, and PANC‐1 cells (IC_50_ values of 9.6, 57.0, and 17.7 μM, respectively) (Figure 5B). Treatment with B02 (10 μM) potentiated the cytotoxic effect of KR12 in KR12‐resistant AsPC‐1 (IC_50_ = 6.8 μM) and PANC‐1 (IC_50_ = 0.2 μM) cells (Figure 5C). Furthermore, we combined siRNA against *KRAS* and treatment with B02 (3 μM) in PANC‐1 cells. We found no significant change in the expression level of *RAD51* after *KRAS* knockdown (Figure S5). As shown in Figure 5D, *KRAS* knockdown significantly suppressed cell proliferation after treatment with B02 versus control siRNA. These data suggested that RAD51 could be a prognostic marker and an important therapeutic target for PDAC.

## 4 DISCUSSION

Although *KRAS* mutations occur early in the development of pancreatic cancer, they are not necessarily involved in disease progression. Previous studies have shown that RNAi‐mediated *KRAS* silencing reduces viability and/or induces apoptosis in PDAC cells although the effect of *KRAS* knockdown is variable between PDAC cell lines.^26^, ^27^, ^28^ It is crucially important to identify a mechanism responsible for the *KRAS* independence in order to develop a clinically effective therapeutic strategy against mutant *KRAS*‐positive PDAC.

In our study, the anti‐tumor effect of KR12 was limited in the xenograft model using KP‐4 cells, although the in vitro studies suggested the sensitivity of KP‐4 cells to KR12. These data imply that another gene may be related to sensitivity to KR12 in PDAC cells harboring the *KRAS* mutation. Microarray analysis using polyamides that do not recognize mutant *KRAS* identified RAD51 as a candidate gene involved in the susceptibility to KR12. RAD51 is a key protein of homologous recombination repair of double‐strand DNA breaks.^29^, ^30^, ^31^ Recent studies have demonstrated that overexpression of RAD51 contributes to tumor cell development, progression, and drug resistance in various types of cancer. Its high expression is associated with poor survival^29^, ^30^, ^31^, ^32^ and 66% of human PDAC tissue specimens showed overexpression of RAD51.^33^ In colorectal cancer, cells with the *KRAS* mutation are highly dependent on RAD51 for survival.^34^ Hence, RAD51 may serve as a potential target for overcoming chemoresistance.^35^ In pancreatic cancer, the role of RAD51 in PDAC cell proliferation and cancer development has rarely been described, although *KRAS* mutation has been reported to increase RAD51 expression.^31^ The present study demonstrated that inhibition of RAD51 suppressed cell proliferation and sensitized PDAC cells to KR12 and *KRAS* knockdown. Our data suggest that the reduced pro‐survival signals by *KRAS* knockdown and impaired DNA repair pathway by RAD51 inhibition might be sufficient to trigger cell death in PDAC cells. The simultaneous inhibition of the target genes *RAD51* and *KRAS* could be important for establishing a therapeutic strategy against PDAC. Further in vivo verification of the anti‐tumor effect of co‐targeting RAD51 and KRAS is required in the future.

To the best of our knowledge, this is the first work to identify a candidate gene responsible for drug sensitivity using alkylating PI polyamides. In recent years, target gene screening methods using RNAi or CRISPR‐Cas9 have been developed. Loss‐of‐function screening by RNAi or CRISPR‐Cas9 libraries has been used to identify a target gene associated with drug response in pancreatic cancer cell lines.^36^, ^37^, ^38^, ^39^ Using the siRNA library directed to all human kinase genes, Giroux et al. reported gemcitabine resistance‐related kinases, including TTK and polo like kinase 1 (PLK1). These genes were also identified by our screening method using alkylating PI polyamides, suggesting their general function in a mechanism of drug sensitivity in PDAC cells. Nevertheless, the *RAD51* has rarely been identified by other screening systems. The present study suggested that the PI polyamide‐based approach may provide a powerful tool for identifying therapeutic target genes associated with drug response in PDAC cells.

Because alkylating PI polyamides can be designed to selectively bind to nine‐base‐pair DNA of the genome following Dervan's recognition rule,^10^ the 9121 target sites were estimated to be potential off‐targets of KR12 in the reference human genome.^11^ However, KR12 is effectively accumulated in tumor tissues owing to its enhanced permeability and retention effect, and exhibits anti‐tumor effects at a low dose without weight loss or adverse effects on other organs.^40^, ^41^, ^42^ Although the low systemic toxicity of intraperitoneally injected KR12 was also observed in the spontaneous PDAC model in this study, further examination is necessary to identify a potential influence of KR12 on normal cells and tissues. To estimate a patient's risk for toxicity, the adverse effects of the combination of KR12 and RAD51 inhibitor also need to be assessed in a future study.

In conclusion, our results suggest that mutant *KRAS*‐targeting KR12 exhibits growth inhibition in a mouse model of spontaneous PDAC. However, its anti‐tumor activity was limited in the human PDAC xenograft mouse model. Screening with PI polyamides identified *RAD51* as a therapeutic target gene involved in drug susceptibility in PDAC. Inhibition of RAD51 combined with KRAS‐targeting drugs is a potential therapeutic strategy for patients with PDAC.

## AUTHOR CONTRIBUTIONS

**Akiko Tsujimoto:** Formal analysis (supporting); investigation (equal); writing – original draft (lead). **Niina Matsuo:** Formal analysis (supporting); investigation (lead). **Xiaoyi Lai:** Formal analysis (equal); investigation (equal). **Takahiro Inoue:** Investigation (supporting); methodology (supporting); supervision (supporting). **Hiroyuki Yoda:** Investigation (supporting); methodology (supporting). **Jason Lin:** Data curation (equal); formal analysis (equal); investigation (equal); software (lead); visualization (supporting); writing – original draft (supporting). **Yoshinao Shinozaki:** Investigation (equal); methodology (equal). **Takayoshi Watanabe:** Investigation (supporting); methodology (supporting). **Nobuko Koshikawa:** Investigation (supporting); methodology (supporting); project administration (supporting). **Atsushi Takatori:** Conceptualization (equal); data curation (lead); formal analysis (lead); funding acquisition (lead); investigation (lead); methodology (equal); project administration (equal); resources (equal); supervision (lead); visualization (lead); writing – original draft (equal); writing – review and editing (lead). **Hiroki Nagase:** Conceptualization (equal); funding acquisition (equal); project administration (equal); resources (equal); supervision (equal); writing – review and editing (supporting).

## FUNDING INFORMATION

This work was supported in part by the Practical Research for Innovative Cancer Control from the Japan Agency for Medical Research and Development (AMED) (grant number JP15ck0106182 to A. Takatori); MEXT KAKENHI (grant numbers JP 25830092 and JP19K07767 to A. Takatori); Takeda Science Foundation (to A. Takatori); Princess Takamatsu Cancer Research Fund (to H. Nagase); JSPS KAKENHI (grant numbers JP20H03540, JP26290060, JP17H03602, and JP16H01579 to H. Nagase); and AMED (grant number 18ae0101051 to H. Nagase).

## CONFLICT OF INTEREST

The authors declare no conflict of interest.

## ETHICS APPROVAL

This study was performed in strict accordance with the recommendations in the Guide for the Care and Use of Laboratory Animals of the Ministry of Education, Culture, Sports, Science and Technology of Japan. The protocol was approved by the Committee on the Ethics of Animal Experiments of Chiba Cancer Center (IRB ID #18–2).

## Supporting information


Figure S1

Figure S2

Figure S3

Figure S4

Figure S5

Table S1

## Data Availability

The microarray data are deposited at the Gene Expression Omnibus (GEO) repository under accession number GSE208243. Other data are available on request from the corresponding author.
