# Supplementary figures and images for: Use of DNA‐alkylating pyrrole‐imidazole polyamides for anti‐cancer drug sensitivity screening in pancreatic ductal adenocarcinoma
Source: Cancer Med. 2022 Oct 19;12(5):5821–32. doi: 10.1002/cam4.5359 (PMC10028039; doi:10.1002/cam4.5359)

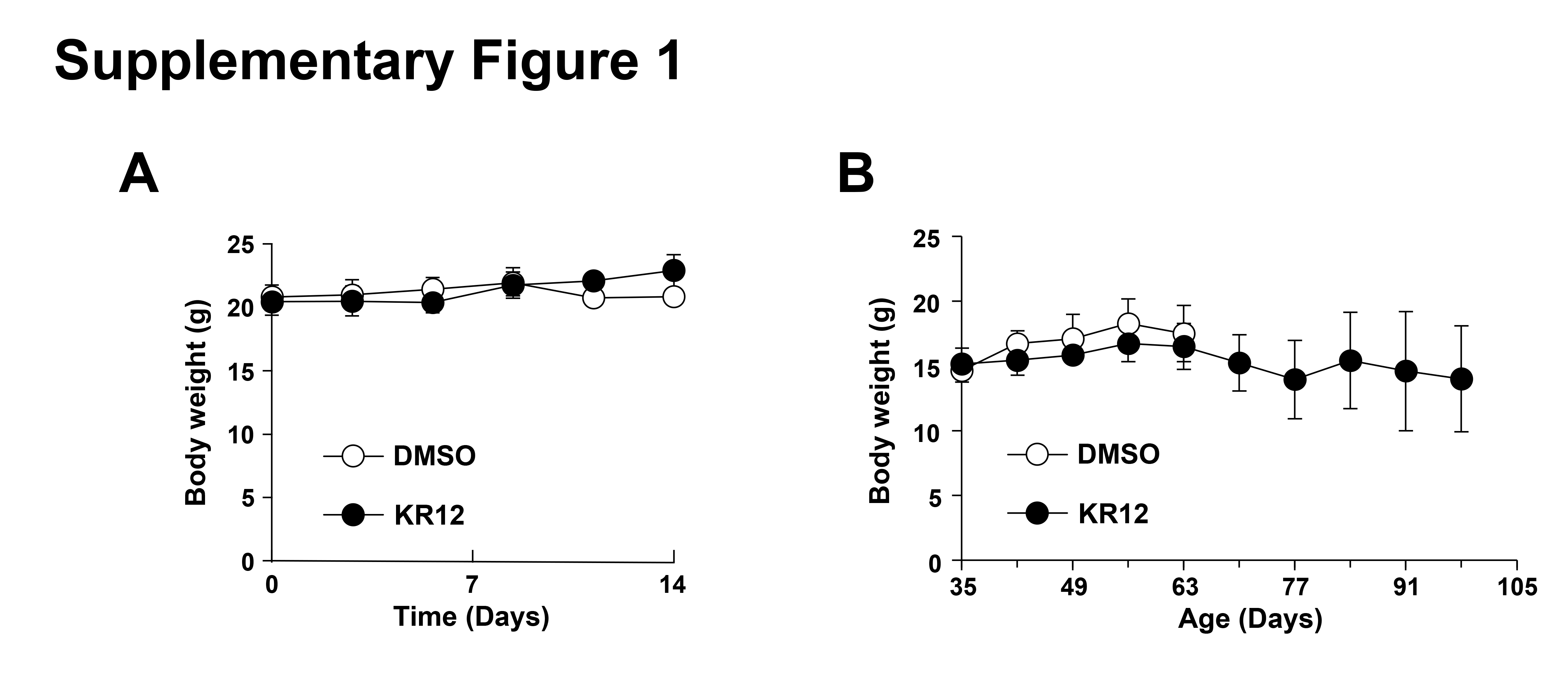

Supplement: Supplementary file 1 — Figure S1 [file CAM4-12-5821-s002.tif]

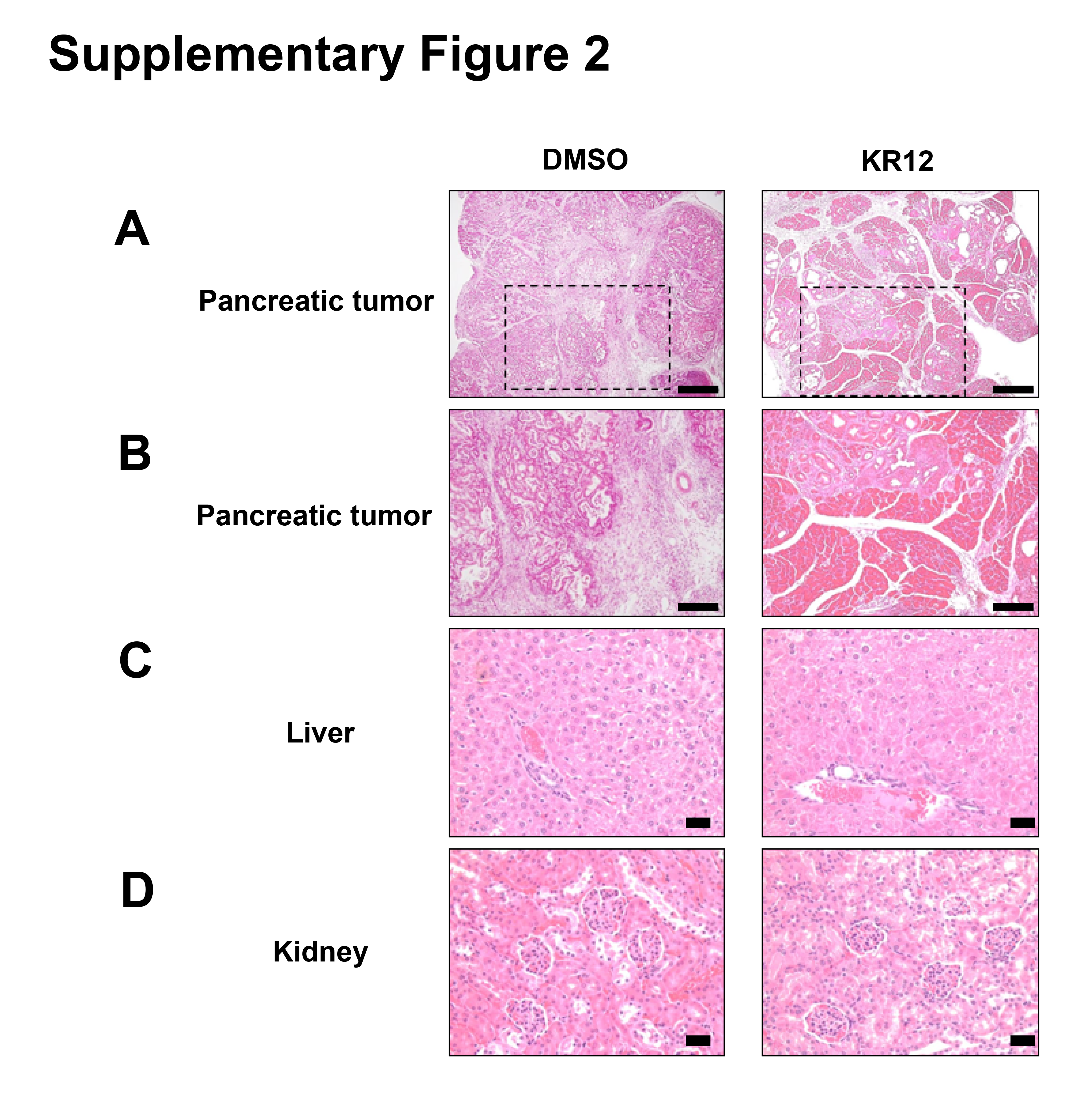

Supplement: Supplementary file 2 — Figure S2 [file CAM4-12-5821-s001.tif]

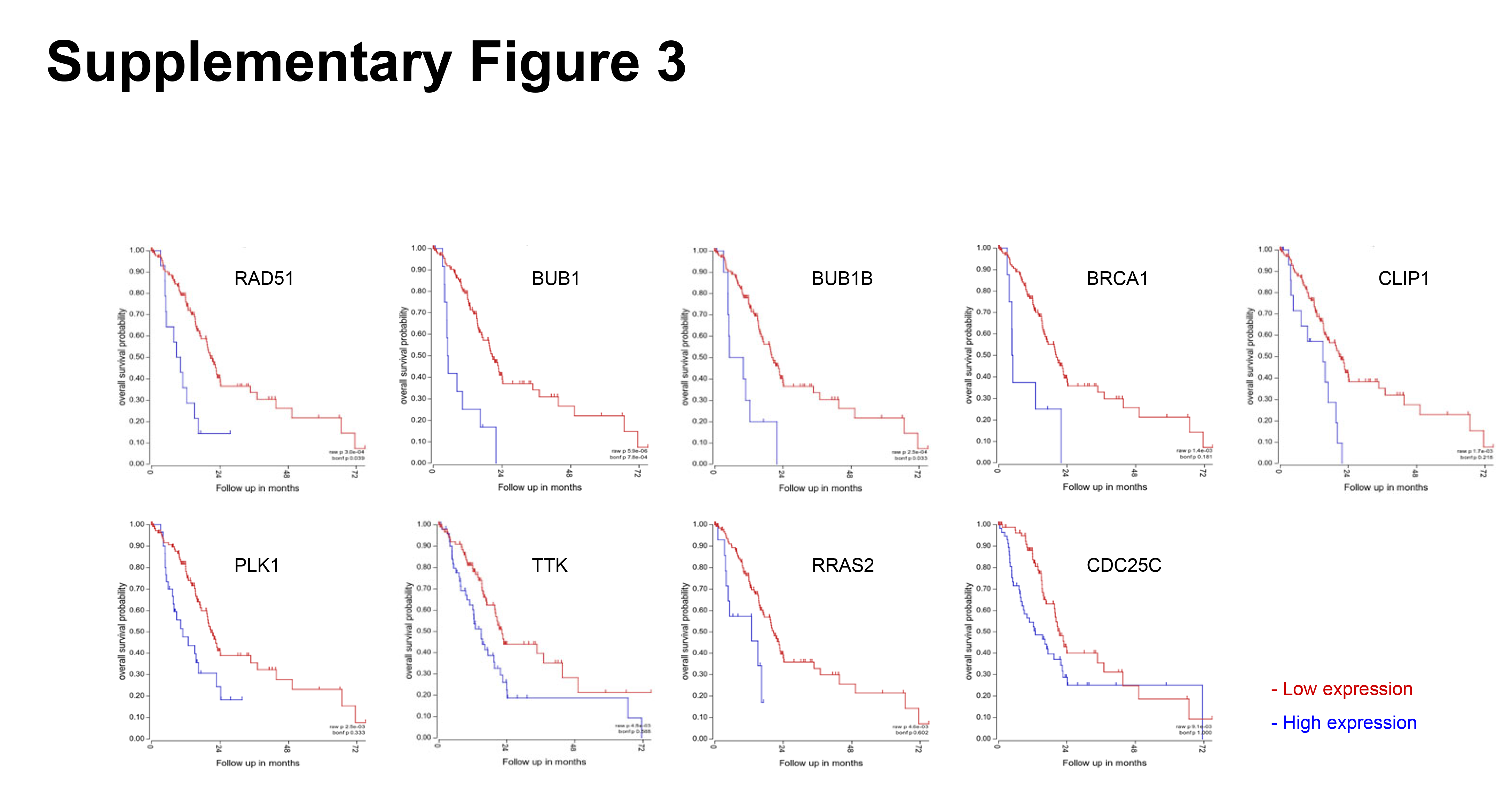

Supplement: Supplementary file 3 — Figure S3 [file CAM4-12-5821-s006.tif]

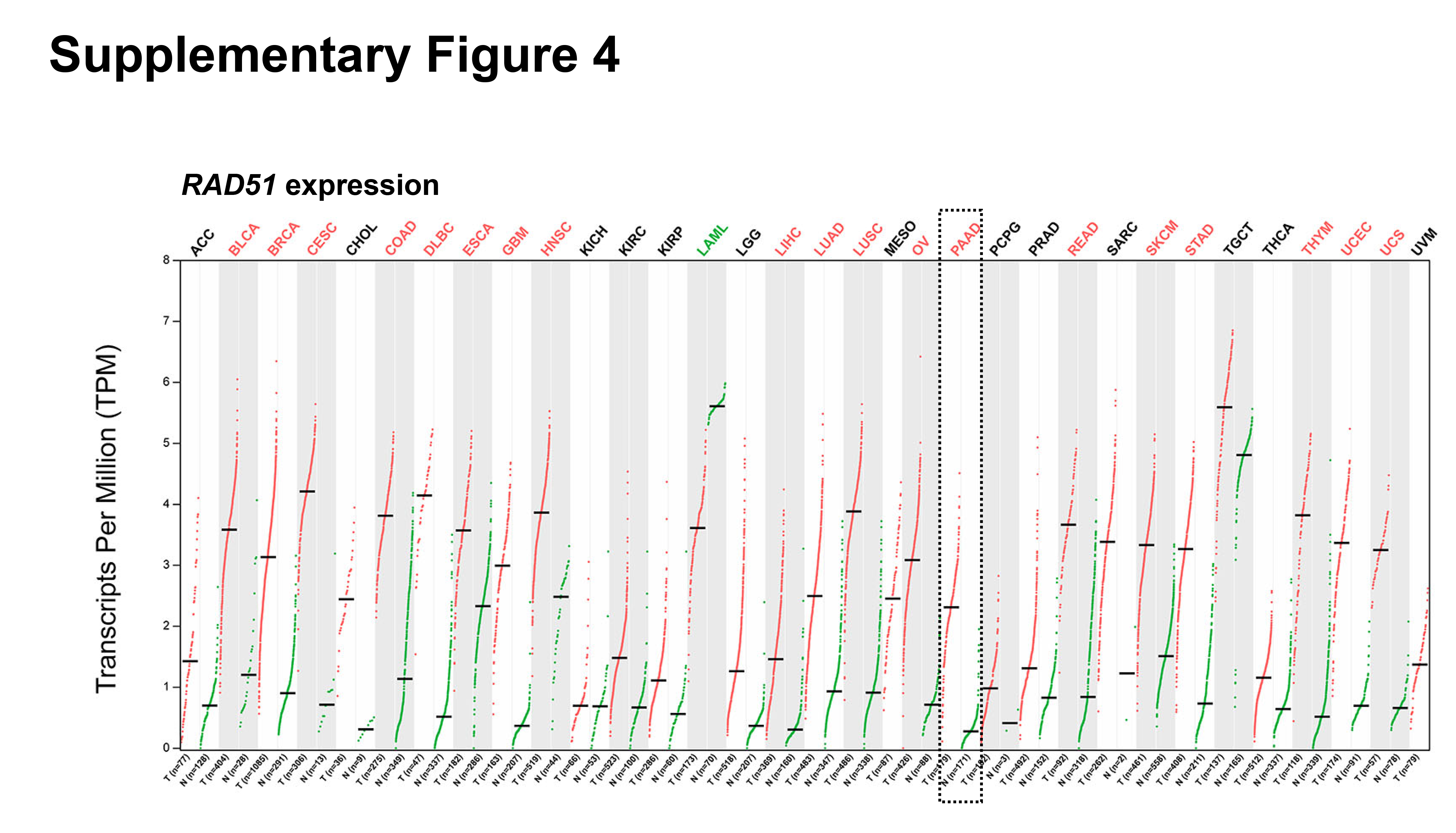

Supplement: Supplementary file 4 — Figure S4 [file CAM4-12-5821-s004.tif]
