# Supplementary material for: Use of DNA‐alkylating pyrrole‐imidazole polyamides for anti‐cancer drug sensitivity screening in pancreatic ductal adenocarcinoma
Source: Cancer Med. 2022 Oct 19;12(5):5821–32. doi: 10.1002/cam4.5359 (PMC10028039; doi:10.1002/cam4.5359)
Supplement: Supplementary file 6 — Table S1 [file CAM4-12-5821-s005.docx]

| Reagent | Supplier | Catalog Number |
| --- | --- | --- |
| Fmoc-β-alanine-OH | Novabiochem | 8. 52024. 0025 |
| Fmoc-γ-Abu-OH | Novabiochem | 8. 52043. 0025 |
| 2-Cl-Trt-Cl resin | Watanabe Chemical Industries | A00330 |
| 1-Ethyl-3-(3-dimethylaminopropyl)carbodiimide hydrochloride (WSCD) | Peptide Institute | 1020 |
| *O*-(6-chlorobenzotriazol-1-yl)-*N*,*N*,*N’*,*N’*-tetramethyluronium hexafluorophosphate  (HCTU) | Peptide Institute | 1047 |
| HPLC-grade acetonitrile | SIGMA | 34888 |
| biotin | SIGMA | B4501 |
| Oxime resin LL (100-200 mesh) | Novabiochem | 8.55089.0005 |
| *N*-methyl-2-pyrrolidone (NMP) | Fujifilm-Wako Chemicals | 131-15111 |
| *N*, *N*-diisopropylethylamine　（DIEA） | Fujifilm-Wako Chemicals | 051-05351 |
| 4-(Fmoc-amino)-1-methyl-1H-pyrrole-2-carboxylic Acid | Fujifilm-Wako Chemicals | 322-64530 |
| 4-(Fmoc-amino)-1-methyl-1H-imidazole-2-carboxylic Acid | Fujifilm-Wako Chemicals | 329-64540 |
| Fmoc-PyIm-COOH | Fujifilm-Wako Chemicals | 289-73361 |
| 1,1,1,3,3,3-Hexafluolo-2-propanol | SIGMA | 105228 |

Supplementary Table 1

Reagents used in compound synthesis
